# Aspirin for primary prevention in patients with high cardiovascular risk: insights from CORE-Thailand registry

**DOI:** 10.1038/s41598-023-41864-1

**Published:** 2023-09-05

**Authors:** Wanwarang Wongcharoen, Nichanan Osataphan, Narawudt Prasertwitayakij, Pannipa Suwannasom, Swangjit Suraamornkul, Wattana Wongtheptian, Siriluck Gunaparn, Wachiranun Sirikul, Arintaya Phrommintikul

**Affiliations:** 1 Division of Cardiology, Department of Internal Medicine, Faculty of Medicine, Chiang Mai University, Chiang Mai, 50200 Thailand; 2 Center for Medical Excellence, Faculty of Medicine, Chiang Mai University, Chiang Mai, Thailand; 3 Faculty of Medicine Vajira Hospital, Navamindradhiraj University, Bangkok, Thailand; 4 Division of Cardiology, Department of Internal Medicine, Chiang Rai Prachanukroh Hospital, Chiang Rai, Thailand; 5 Department of Community Medicine, Faculty of Medicine, Chiang Mai University, Chiang Mai, Thailand

**Keywords:** Cardiology, Risk factors

## Abstract

Aspirin may be considered for primary prevention in non-elderly patients with high cardiovascular risk. However, contemporary management aimed at aggressive cardiovascular risk factor control may alter benefit-risk ratio of aspirin. Therefore, we aimed to examine the effect of aspirin for primary prevention on the long-term MACEs in a large cohort registry. Cohort Of patients with high Risk for cardiovascular Events (CORE-Thailand) registry is a prospective, multicenter, observational, longitudinal study of Thai patients with high atherosclerotic risk. Patients with established atherosclerotic cardiovascular diseases were excluded. Among 4259 patients with multiple cardiovascular risk factors, 1945 (45.7%) patients used aspirin. After propensity score matching, there were 3228 patients remained in post-matching analysis. During the median follow-up period of 58.2 months, we demonstrated that aspirin use increased risk of long-term MACEs in pre-matching cohort (unadjusted HR 1.76, 95% CI 1.43–2.17, *P* < 0.001) and post-matching cohort (HR 1.66 (1.31–2.10), *P* < 0.001). In addition, patients taking aspirin had a higher risk of bleeding than non-aspirin users in pre-matching cohort (unadjusted HR 2.28, 95% CI 1.09–4.75, *P* = 0.028). We demonstrated that aspirin was associated with increased risk of long-term MACEs in patients with multiple cardiovascular risk factors. Due to the non-randomized design, our results should be interpreted with caution.

## Introduction

The benefit of low-dose aspirin is well-established for secondary prevention in patients with established atherosclerotic cardiovascular diseases (ASCVD)^1^. Nevertheless, the advantage of aspirin for primary prevention is far less pronounced. The ASPREE (Aspirin in Reducing Events in the Elderly) trial showed that aspirin for primary prevention did not reduce risk of major cardiovascular events (MACEs) but increased risk of major bleeding and total mortality in elderly population^2,3^. The ARRIVE (Use of Aspirin to Reduce Risk of Initial Vascular Events in Patients at Moderate Risk of Cardiovascular Disease) study demonstrated the lack of aspirin’s benefit for primary prevention in subjects with intermediate risk for ASCVD^4^. Only those with diabetes mellitus (DM) were demonstrated to benefit from aspirin for primary prevention in the ASCEND (A Research of Cardiovascular Events in Diabetes) study. However, the increased risk of serious hemorrhage diminished the value of aspirin^5^.

Despite the negative results of aspirin from several randomized controlled trials (RCTs), the recent meta-analysis comprised of 11 RCTs and 1 pilot trial has shown that low-dose aspirin reduced incidence of MACEs including myocardial infarction and stroke. Nevertheless, aspirin increased risk of significant bleeding and did not decrease the cardiovascular mortality or all-cause mortality^6^. Based on the available data, international guidelines advised against the routine use of aspirin in individuals over the age of 70 or those who had a high bleeding risk. The comprehensive shared decision-making is suggested by the guidelines in healthy patients aged below 70 years when considering aspirin for primary prevention^7–9^.

The presented evidence suggested that aspirin may have benefit in selected population such as those with diabetes and those with high cardiovascular risk. However, contemporary management aimed at aggressive cardiovascular risk factor control, as well as increased statin use in this high-risk population, may alter the benefit-risk ratio of aspirin demonstrated in previous trials. Therefore, we aimed to examine the effect of aspirin for primary prevention on the long-term MACEs in a large cohort registry of high atherosclerotic risk patients in Thailand. The differential effects of aspirin in distinct subgroups were also investigated.

## Methods

### Study population

The Cohort Of patients with high Risk for cardiovascular Events (CORE-Thailand) registry is a prospective, multicenter, observational, longitudinal study of Thai patients with high atherosclerotic risk. The detail of the CORE-Thailand registry has been published previously^10^. The study was approved by the Joint Research Ethics Committee and Ministry of Public Health, Thailand. Informed consent was obtained from all patients prior to the commencement of the study.

The whole cohort of CORE-Thailand registry comprised of patients aged ≥ 45 years with established CAD, stroke /transient ischemic attack (TIA), or peripheral arterial disease (PAD), or patients with multiple cardiovascular risk factors from the outpatient clinics from April 2011 to March 2014. The multiple cardiovascular risk factors were defined as the presence of at least three atherosclerosis risk factors, including male > 55 years, female > 65 years, diabetes mellitus (DM) or impaired fasting glucose, hypertension, dyslipidemia, chronic kidney disease (CKD) defined as presence of proteinuria + 1 or eGFR < 60 ml/min) and family history of premature ASCVD. As we aimed to study the effect of aspirin for primary prevention, we excluded patients with established cardiovascular diseases from our analysis and included only patients with multiple risk factors. Patients who received antiplatelet medications other than aspirin or chronic anticoagulation therapy (> 3 months) were also excluded. The median follow-up period was 58.2 months (IQR 34.8, 60.9 months).

### Data collection

Baseline demographic data, cardiovascular risk factors, co-morbidities, medication at the time of enrollment and laboratory data were collected in all patients. Data were locally collected using a standardized case report form. Patient data was then forwarded to the data management group of the Medical Research Network of the Consortium of Thai Medical Schools (MedResNet). Prior to data analysis, data quality and completeness were evaluated. A yearly random site monitoring was conducted.

### The study endpoints

The participants with multiple cardiovascular risk factors were followed for the first occurrence of MACEs until March 2019. The primary endpoint was the effect of aspirin on long-term MACEs which were defined as a composite outcome of nonfatal myocardial infarction, nonfatal stroke, and death from any cause. The secondary outcome was the effect of aspirin on each individual outcome. In addition, the effect of aspirin on bleeding events was also explored.

### Statistical analysis

Continuous variables were represented as either means and standard deviations when they followed a normal distribution, or as medians and interquartile ranges when the distribution was not normal. To compare variations among the distinct groups, the Mann–Whitney U test or the Student t-test was employed. Categorical variables were displayed as frequencies (%) and their comparison between groups was conducted using Fisher's exact test. The cumulative incidence of the primary outcome was computed using Kaplan–Meier curves, and the log-rank test was utilized to determine group differences. The risk of MACEs was compared using Cox regression analysis and was reported as hazard ratio (HR) with 95% confidence interval (CI). For multivariable adjustment, variables of age, gender, baseline comorbidities, and medications were retained. In addition, we adjusted for variations in baseline characteristics by producing a propensity-score approach analyses including propensity score adjustment, propensity score stratification, propensity score matching and inverse probability treatment weighting (IPTW). The propensity score matching was conducted using the caliper width of 0.2 standard deviation (SD). This allowed further reduction of selection bias while maintaining confounding variables evenly distributed across the two groups (Fig S1). After matching, we reassessed the baseline characteristics to account for any remaining confounding. We calculated the standardized difference (Std) to evaluate the balance; a Std value > 0.03 was considered indicative of a notable difference and led us to include those variables for further confounding adjustment^11^. Subsequently, we conducted a multivariable analysis (double adjustment after propensity score matching) to mitigate the influence of any remaining confounding factors. We also performed subgroup analysis in the propensity-score matched population. In the matched population, we conducted stratified Cox regression analysis to compare mortality and subgroup analysis to reveal any interaction between the observed association and relevant factors Statistical significance was established as a *P*-value below 0.05. The statistical analysis was performed using the SPSS software package version 23 (IBM Corp., Armonk, NY, USA, https://www.ibm.com/products/spss-statistics), while graphic visualization was accomplished using STATA version 16.2 (StataCorp, College Station, TX, USA, https://www.stata.com/order/new/edu/profplus/campus-profplus/).

### Ethics approval and consent to participate

This study was approved by the Joint Research Ethics Committee and Ministry of Public Health, Thailand (Certificate Number COA-JREC 004/2011). Informed consent was obtained from all patients prior to the commencement of the study and was registered in thaiclinicaltrials.org, identification number TCTR20130520001. The investigations were carried out following the Declaration of Helsinki, including written informed consent from all participants.

## Results

**Figure 1:**
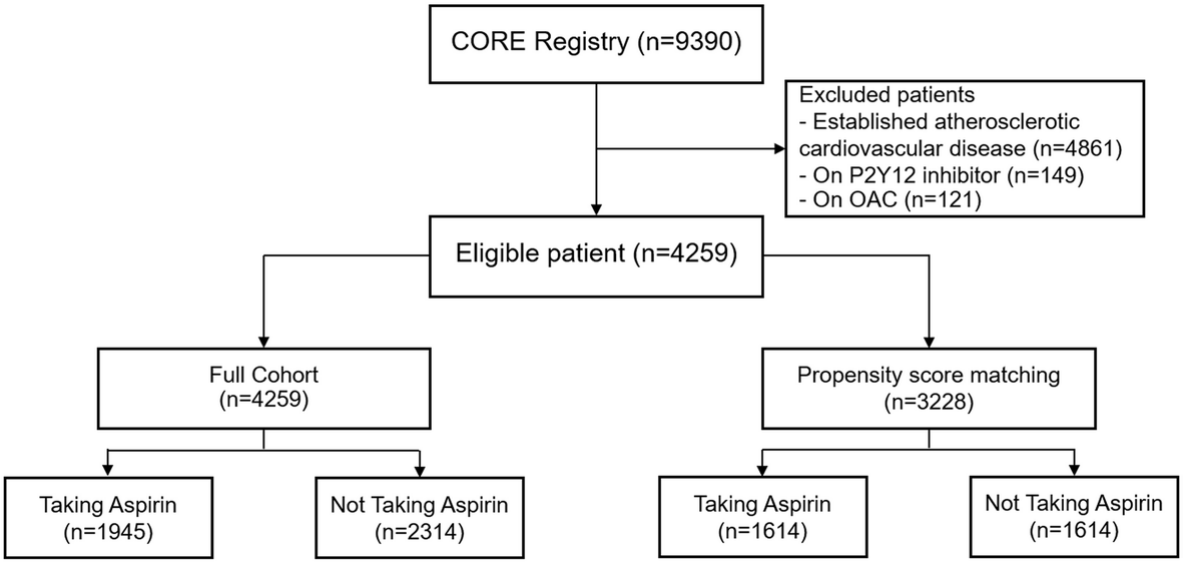
Consort diagram.

A total of 9390 patients with established ASCVD or multiple cardiovascular risk factors were enrolled in CORE-Thailand registry. There were 4861 patients with established ASCVD which were excluded from the analysis. The 149 patients taking antiplatelet drug other than aspirin and 121 patients taking oral anticoagulants were also excluded from the study. In the final analysis, 4259 eligible patients were included (Fig. 1).

### The patient characteristics

Among 4259 patients with multiple cardiovascular risk factors, 1945 (45.7%) patients used aspirin. The baseline characteristics between patients with and without aspirin use are shown in Table 1. The mean age was 65.4 ± 9.5 years which was comparable between patient with and without aspirin use. The baseline blood pressure (BP) was similar between the 2 groups. The mean systolic and diastolic BP was 134.9 ± 16.9/75.4 ± 10.7 mmHg. Higher proportion of male patients was demonstrated in aspirin users compared to non-aspirin users (47.2% vs. 38.1%, *P* < 0.001). The patients taking aspirin had a higher prevalence of DM, metabolic syndrome, hypertension and CKD. However, the family history of premature ASCVD was reported more frequently in non-aspirin user. The greater use of beta-blocker, angiotensin converting enzyme inhibitor or angiotensin receptor blocker and statin was demonstrated in patients taking aspirin. The median duration of aspirin therapy in aspirin users was 46 months (IQR 24, 60 months).

**Table 1:** Baseline characteristics between patients with and without aspirin use.

	Full cohort (n = 4259)				Propensity score matching (n = 3228)			
	Aspirin	No Aspirin	*P*-value	Std diff	Aspirin	No Aspirin	*P*-value	Std diff
	(n = 1945)	(n = 2314)			(n = 1614)	(n = 1614)		
Age (years)	65.32 ± 9.47	65.45 ± 9.62	0.66	0.014	65.25 ± 9.53)	65.07 ± 9.61	0.59	− 0.019
Male (%)	918 (47.20)	881 (38.10)	< 0.01	0.185	675 (41.82)	674 (41.76)	0.97	0.001
Body mass index (kg/m^2^)	26.49 ± 4.46	25.73 ± 4.37	< 0.01	− 0.172	26.24 ± 4.40	26.16 ± 4.59	0.63	− 0.170
Systolic blood pressure (mmHg)	135.18 ± 17.46	134.59 ± 16.39	0.26	− 0.034	135.16 ± 17.27	135.03 ± 16.30	0.83	− 0.008
Diastolic blood pressure (mmHg)	75.49 ± 10.83	75.26 ± 10.57	0.49	− 0.021	75.57 ± 10.82	75.62 ± 10.50	0.89	0.004
Medical history								
Diabetes mellitus (%)	1611 (82.80)	1614 (69.75)	< 0.01	0.311	1290 (79.93)	1312 (81.29)	0.33	0.034
Metabolic syndrome (%)	1347 (69.25)	1288 (55.66)	< 0.01	0.283	1062 (65.80)	1058 (65.55)	0.88	0.005
Hypertension (%)	1892 (97.30)	2156 (93.20)	< 0.01	0.193	1561 (96.72)	1559 (96.59)	0.84	0.007
Chronic kidney disease (%)	567 (29.20)	489 (21.13)	< 0.01	0.185	421 (26.08)	396 (24.54)	0.31	0.036
Dyslipidaemia (%)	1821 (93.62)	2171 (93.82)	0.801	0.008	1513 (93.74)	1521(94.24)	0.55	0.021
Current smoke (%)	85 (4.40)	82 (3.50)	0.18	0.042	53 (3.28)	58 (3.59)	0.63	0.017
Family history of atherosclerosis (%)	106 (5.40)	189 (8.20)	< 0.01	0.108	97 (6.01)	96 (5.95)	0.94	0.003
Medication								
Beta-blocker (%)	730 (37.50)	674 (29.10)	< 0.01	0.179	536 (33.21)	544 (33.71)	0.77	0.010
Angiotensin-converting enzyme inhibitors/angiotensin receptor blockers (%)	1403 (72.10)	1484 (64.10)	< 0.01	0.172	1150 (71.25)	1152 (71.38)	0.94	0.003
Statin (%)	1694 (87.10)	1880 (81.20)	< 0.01	0.160	1379 (85.44)	1369 (84.82)	0.62	0.017

Std diff; standardized difference.

We performed the propensity score matching to eliminate the potential confounding factors between aspirin user and non-aspirin user. After propensity score matching, there were 3228 patients remained in the post-matching analysis, which 1614 patients were in each group. The post-matching analysis showed no difference in baseline characteristics, co-morbidities and medications between aspirin users and non-aspirin users in term of *p*-value. (Table 1) The standardized difference above 0.03 was observed in BMI, diabetes mellitus and chronic kidney disease. These variables were incorporated for adjustment after propensity score matching.

### The effect of aspirin on the study outcomes

**Figure 2:**
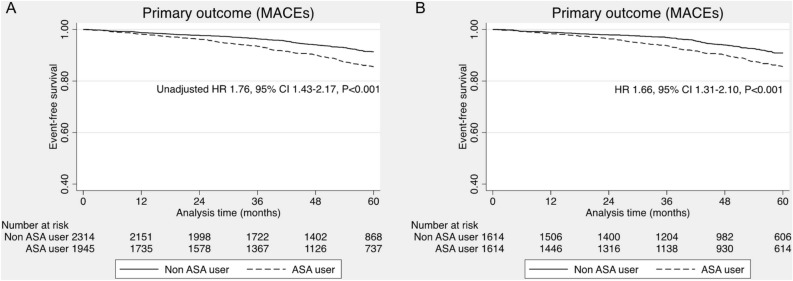
Primary outcome of major cardiovascular events. (**A**) Full cohort, (**B**) Propensity score matching.

**Figure 3:**
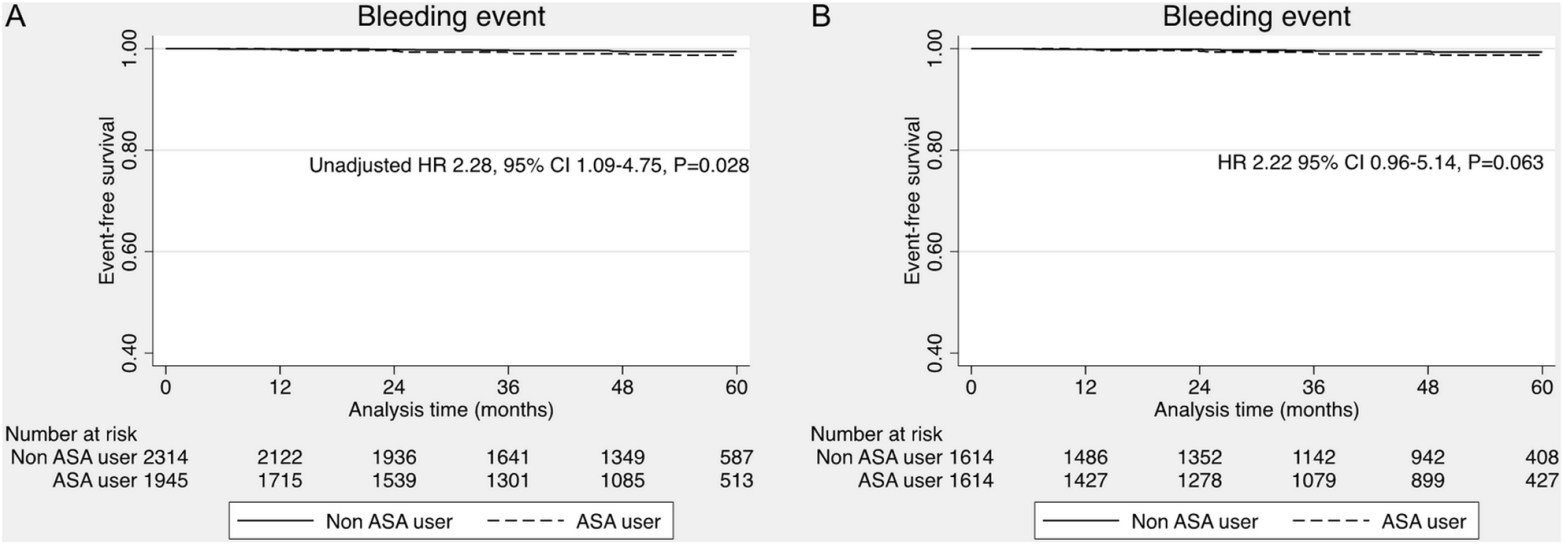
Bleeding events. (**A**) Full cohort, (**B**) Propensity score matching.

During the median follow-up period of 58.2 months, we demonstrated that use of aspirin increased risk of the long-term MACEs (unadjusted HR 1.76, 95% CI 1.43–2.17, *P* < 0.001) in the pre-matching cohort of 4259 patients (Fig. 2). Further analysis with propensity score adjustment, propensity score stratification, propensity score weighing (IPTW), stabilized propensity score weighing (stabilized IPTW) and covariables matching showed consistent results, indicating that taking aspirin increased the risk of MACEs (Table 2). The higher incidence of all-cause mortality was also present in patients taking aspirin than non-aspirin users (unadjusted HR 1.62, 95% CI 1.30–2.03, *P* < 0.001). (Table 2, Fig. 2) In addition, patients taking aspirin had a higher risk of bleeding than non-aspirin users (unadjusted HR 2.28, 95% CI 1.09–4.75, *P* = 0.028) (Fig. 3).

**Table 2:** Primary outcome in full cohort, covariable matching and propensity score matching.

Primary outcome	HR (95% CI)	*P*-value
Full cohort (N = 4205)		
Univariable	1.76 (1.43–2.17)	< 0.001
Multivariable*	1.47 (1.16–1.89)	< 0.001
Propensity score adjustment	1.55 (1.25–1.93)	< 0.001
Propensity score stratification	1.59 (1.28–1.97)	< 0.001
Propensity score weighing (IPTW)	1.53 (1.23–1.91)	< 0.001
Stabilized propensity score weighing (stabilized IPTW)	1.53 (1.23–1.91)	< 0.001
Covariables matching (N = 3206)		
Covariables matching^$^	1.67 (1.31–2.14)	< 0.001
Propensity score matching (N = 3228)		
Propensity score matching	1.66 (1.31–2.10)	< 0.001
Propensity score matching with double adjustment of residual confounders^#^	1.62 (1.27–2.05)	< 0.001

*Adjusted with age, sex, BMI, history of smoking, systolic blood pressure, diastolic blood pressure, hypertension, diabetes mellitus, chronic kidney disease, dyslipidemia, beta-blocker, Angiotensin-converting enzyme inhibitors/ Angiotensin receptor blockers, and statin.

^$^Covariables including sex, hypertension, diabetes mellitus, chronic kidney disease, dyslipidemia, family history of atherosclerosis, beta-blocker, Angiotensin-converting enzyme inhibitors/ Angiotensin receptor blockers, and statin.

^#^Adjusted with BMI and metabolic syndrome.

After propensity score matching with potential covariates, the use of aspirin remained an independent risk factor for the occurrence of long-term MACEs (HR 1.66, 95% CI 1.31–2.10, *P* < 0.001) (Table 2, Fig. 2). In terms of secondary outcomes, the patients taking aspirin were associated with increased risk of all-cause mortality (HR 1.53, 95% CI 1.18–1.97, *P* = 0.001). Furthermore, the greater risk of fatal and non-fatal myocardial infarction was noted in aspirin users (HR 3.93, 95% CI 1.30–11.80, *P* = 0.015). The risk of fatal and non-fatal stroke tended to increase in aspirin users as compared to non-aspirin users (HR 1.76, 95% CI 0.99–3.11, *P* = 0.05). Nevertheless, the use of aspirin did not significantly increase risk of bleeding in post-matching cohort (HR 2.22, 95% CI 0.96–5.14, *P* = 0.063) (Fig. 3).

**Figure 4:**
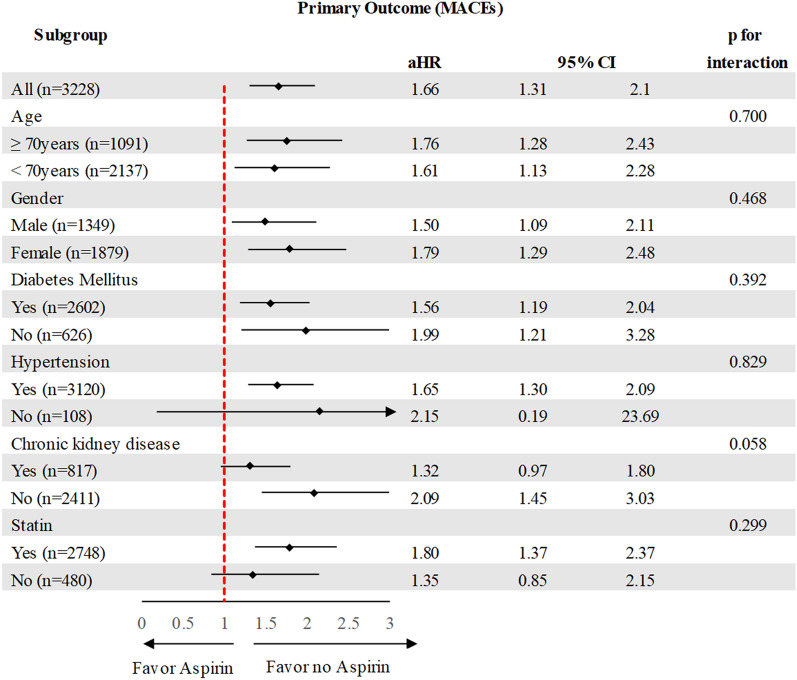
Subgroup analysis of primary outcome in the propensity score matching cohort.

The subgroup analysis demonstrated that aspirin increased risk of long-term MACEs in patients across the various subgroups (Fig. 4).

## Discussion

There are some heterogeneities between guideline recommendations for the use of aspirin in primary prevention of cardiovascular disease. The 2019 American Heart Association/American College of Cardiology guidelines suggested that persons aged 40–70 years at elevated cardiovascular risk may be considered to take aspirin for the primary prevention of cardiovascular disease^8^. However, 2021 European guidelines gave a class IIb recommendation for aspirin only in diabetic patients at high cardiovascular risk. They suggested personalized decision-making when deciding whether to provide aspirin to healthy subjects under the age of 70 who were at high cardiovascular risk^7^. The American Diabetes Association advised that, following a collaborative decision-making process, low-dose aspirin be taken into consideration for diabetic patients with higher ASCVD risk and low bleeding risk^9^. Importantly, both the American and European guidelines advised against the routine use of aspirin in individuals over the age of 70 or those who had a high bleeding risk^7,8^. Recently, US Preventive Services Task Force (USPSTF) has downgraded the recommendation of aspirin for primary prevention in patients aged 40–59 years with 10-year cardiovascular risk ≥ 10% from grade B (offer or provide this service) to grade C (might be considered). In addition, the USPSTF no longer advises persons 60 years of age or older to start taking low-dose aspirin^12^. Collectively, the international guidelines do not support the routine use of aspirin for primary prevention. Nevertheless, they stated that aspirin may be considered for primary prevention in non-elderly patients with high cardiovascular risk. However, the benefit of aspirin in this population is still inconclusive.

Recently, the observational studies revealed the lack of benefit and possibly harmful effect of aspirin for primary prevention in patients at high risk for cardiovascular diseases. One retrospective cohort study found that the patients with CKD without previous cardiovascular disease did not receive benefit from long-term aspirin therapy^13,14^. Another cohort study explored the outcome of aspirin in hypertensive patients with obstructive sleep apnea (OSA). The investigators demonstrated that long-term use of aspirin was associated with the increased risk of MACEs in this population^15^. Similar to our results, we demonstrated that aspirin was associated with the higher risk of long-term MACEs in patients with multiple cardiovascular risk factors.

Furthermore, our findings were in agreement with previous study examining the effect of aspirin in patients with non-obstructive coronary artery disease (CAD) determined by coronary computed tomography angiography^16^. They demonstrated that patients with non-obstructive CAD was associated with the increased risk of MACEs and all-cause mortality compared to those with no detectable plaque Nevertheless, aspirin did not reduce risk of MACEs and mortality but the use of statin was associated with the lower risk of MACEs in this population.

There have been conflicting data regarding aspirin among randomized trials in primary prevention, possibly due to variations in the risk profiles of the studied populations^2,3,17^. The JPAD (Japanese Primary Prevention of Atherosclerosis With Aspirin for Diabetes) study demonstrated that aspirin did not increase the risk of either MACEs or major bleeding in diabetic patients^17^. In contrast, the ASPREE study showed that elderly patients taking aspirin had a higher risk of all-cause mortality and major bleeding compared to those taking a placebo^2,3^. This finding aligns with our results. The population studied in the ASPREE trial was older and had a higher risk profile than the population in the JPAD study, which could account for the observed increased risk of bleeding and all-cause mortality. While our results were similar to those of the ASPREE study, they differed from those of the JPAD study. Although a majority of our patients had diabetes, similar to the JPAD study, our study noted a higher prevalence of hypertension and CKD in comparison to the JPAD study. Furthermore, in our study, statins were prescribed to over 80% of the patients, a percentage notably higher than that reported in other randomized trials evaluating aspirin for primary prevention. Specifically, the utilization of statins was observed in only 26%, 34%, 43%, and 75% of the participants in JPAD^17^, ASPREE^2^, ARRIVE^4^, and ASCEND^5^ trials, respectively. The contemporary strategies for cardiovascular risk reduction, which encompass the widespread implementation of statin therapy and the pursuit of more aggressive targets for cholesterol and blood pressure reduction, may potentially overshadow the efficacy of aspirin as a primary preventive measure.

Nevertheless, the reason behind the association between aspirin use and an elevated risk of stroke and myocardial infarction in our study, as well as in other primary prevention studies^15,16^, remains uncertain. Numerous potential explanations have been suggested. In the full cohort, we demonstrated that patients taking aspirin had a higher risk of bleeding than non-aspirin users, which is consistent with findings from previous studies^2,4,18^. The occurrence of major bleeding associated with the use of aspirin could lead to hypotension, anemia, compromised oxygen delivery, vasoconstriction, and platelet dysfunction, all of which might contribute to the heightened risk of myocardial infarction. In addition, the increased bleeding risk in aspirin users may have elevated the risk of hemorrhagic stroke. Furthermore, the presence of a significant bleeding event could exert substantial hemodynamic effects, possibly destabilizing a patient who was already vulnerable in terms of cerebral functions and potentially leading to an increased risk of stroke. Of note, even with the implementation of propensity score matching to mitigate confounding factors, it remains unfeasible to entirely eliminate unmeasurable confounders. It is plausible that patients prescribed aspirin might have possessed a higher risk of ASCVD compared to those who were not prescribed the medication.

Despite the questionable benefit of low-dose aspirin for primary prevention and potential risk of aged population, aspirin has still been prescribed in a sizable portion of patients. A recent survey demonstrated that nearly half of internists reported prescribing aspirin for primary prevention in patients aged above 70 years who had significant cardiovascular risk^19^. Our results demonstrated the potential harm of aspirin for primary prevention irrespective of age and co-morbidities. As a result, aspirin should be discouraged for primary prevention even in patients at high risk for cardiovascular diseases.

The major limitation of our study was inherent to the non-randomized design. To account for confounders and selection bias, we employed multivariate models and propensity score matching. Nevertheless, the unmeasurable confounders cannot be completely excluded. The non-randomized design may introduce systematic biases that could confound the association between aspirin use and increased cardiovascular risk. Consequently, causality may not be estimated correctly. Therefore, our results should be interpreted with caution. The subgroup analysis was conducted post hoc to explore the impact of aspirin within specific subgroups and the sample size might not be sufficient to attain a statistically significant difference within the subgroup (Table S1). The outcomes derived from the subgroup analysis served to generate hypotheses and should be approached with careful interpretation. This study is prospective in nature, and it's worth noting that certain patients died either at home or in their local hospital. In these cases, determining the exact cause of death was not always straightforward. Consequently, we chose to utilize all-cause mortality as the primary outcome of our study. This decision led to our inability to evaluate the impact of aspirin on specific outcomes such as cardiovascular mortality or fatal bleeding.

## Conclusion

We demonstrated that aspirin was associated with the increased risk of long-term MACEs and all-cause mortality in a large cohort study of Thai patients with multiple cardiovascular risk factors. Due to the non-randomized design, our results should be interpreted with caution.

### Supplementary Information

Supplementary Figure S1.

Supplementary Table S1.

## Data Availability

The data that support the findings of this study are available from the corresponding author upon reasonable request.
