# Supplementary figures and images for: Aspirin for primary prevention in patients with high cardiovascular risk: insights from CORE-Thailand registry
Source: Sci Rep. 2023 Sep 5;13:14646. doi: 10.1038/s41598-023-41864-1 (PMC10480154; doi:10.1038/s41598-023-41864-1)

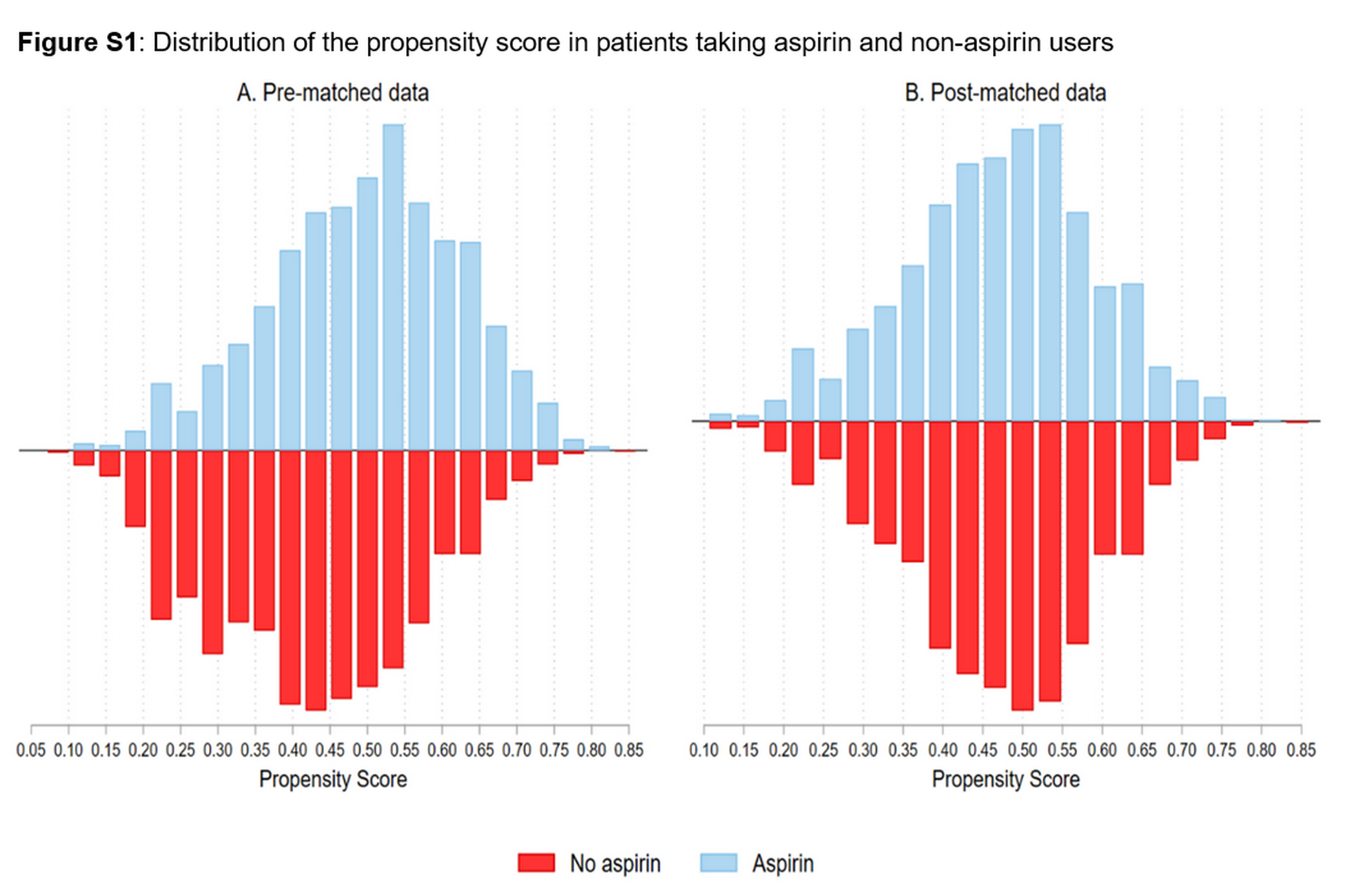

Supplement: Supplementary file 1 — Supplementary Figure S1. [file 41598_2023_41864_MOESM1_ESM.tif]
