# Supplementary material for: Aspirin for primary prevention in patients with high cardiovascular risk: insights from CORE-Thailand registry
Source: Sci Rep. 2023 Sep 5;13:14646. doi: 10.1038/s41598-023-41864-1 (PMC10480154; doi:10.1038/s41598-023-41864-1)
Supplement: Supplementary file 2 — Supplementary Table S1. [file 41598_2023_41864_MOESM2_ESM.docx]

**Table S1** Calculated power for statistical analysis according to the hazard ratio.

| **alpha** | **power** | **N** | **Events** | **delta** | **HR** | **SD** | **Event probability** |
| --- | --- | --- | --- | --- | --- | --- | --- |
| 0.05 | 0.8 | 10,613 | 945 | 0.1823 | 1.2 | 0.5 | 0.089 |
| 0.05 | 0.8 | 5,125 | 457 | 0.2624 | 1.3 | 0.5 | 0.089 |
| 0.05 | 0.8 | 3,116 | 278 | 0.3365 | 1.4 | 0.5 | 0.089 |
| 0.05 | 0.8 | 2,146 | 191 | 0.4055 | 1.5 | 0.5 | 0.089 |
| 0.05 | 0.8 | 1,597 | 143 | 0.47 | 1.6 | 0.5 | 0.089 |
| 0.05 | 0.8 | 1,253 | 112 | 0.5306 | 1.7 | 0.5 | 0.089 |
| 0.05 | 0.8 | 1,022 | 91 | 0.5878 | 1.8 | 0.5 | 0.089 |
| 0.05 | 0.8 | 857 | 77 | 0.6419 | 1.9 | 0.5 | 0.089 |
| 0.05 | 0.8 | 735 | 66 | 0.6931 | 2 | 0.5 | 0.089 |
| 0.05 | 0.8 | 641 | 58 | 0.7419 | 2.1 | 0.5 | 0.089 |
| 0.05 | 0.8 | 568 | 51 | 0.7885 | 2.2 | 0.5 | 0.089 |
| 0.05 | 0.8 | 509 | 46 | 0.8329 | 2.3 | 0.5 | 0.089 |
| 0.05 | 0.8 | 461 | 41 | 0.8755 | 2.4 | 0.5 | 0.089 |
| 0.05 | 0.8 | 421 | 38 | 0.9163 | 2.5 | 0.5 | 0.089 |
| 0.05 | 0.8 | 387 | 35 | 0.9555 | 2.6 | 0.5 | 0.089 |
| 0.05 | 0.8 | 358 | 32 | 0.9933 | 2.7 | 0.5 | 0.089 |
| 0.05 | 0.8 | 333 | 30 | 1.03 | 2.8 | 0.5 | 0.089 |
| 0.05 | 0.8 | 312 | 28 | 1.065 | 2.9 | 0.5 | 0.089 |
| 0.05 | 0.8 | 293 | 27 | 1.099 | 3 | 0.5 | 0.089 |
